# Protecting work engagement from digital fatigue: the contingent roles of leadership style and network ties

**DOI:** 10.3389/fpsyg.2025.1645057

**Published:** 2025-11-14

**Authors:** Xiaozhu Zhang, Guangkuan Deng

**Affiliations:** 1 Chengdu Academy of Social Sciences, Chengdu, China; 2 School of Economics and Management, Southwest University of Science and Technology, Mianyang, China

**Keywords:** digital fatigue, work engagement, leadership style, network ties, JD-R, COR

## Abstract

The growing digitalization of work has reshaped employee experiences, introducing new forms of strain such as digital fatigue—a state of cognitive and emotional exhaustion resulting from excessive digital demands. Drawing on Conservation of Resources (COR) theory and the Job Demands–Resources (JD-R) framework, this study investigates how digital fatigue influences work engagement and how contextual resources—leadership style and network ties—moderate this relationship. Using survey data from 339 employees in Chinese technology firms, the results show that digital fatigue significantly reduces work engagement. Transformational leadership weakens this negative relationship, while transactional leadership strengthens it. In addition, internal network ties mitigate the adverse effect of digital fatigue, whereas external ties have no significant impact. This research deepens the understanding of digital fatigue in digitalized work environments, leadership, and organizational networks, offering insights for managers to adopt transformational leadership and strengthen internal social resources to sustain employee engagement under growing digital demands.

## 1 Introduction

In the digital age, the rapid adoption of technologies has significantly transformed organizational operations and employee engagement (Cavicchioli et al., 2025; Duan et al., 2024; Zhang and Chen, 2023). Digital tools are increasingly integrated into everyday tasks, enabling real-time communication, data-driven decision-making, and flexible work arrangements (Opland et al., 2022). These advancements have undoubtedly boosted productivity and facilitated the remote work revolution (Nucci et al., 2023). However, while digital technologies have enhanced efficiency, they have also introduced new challenges (Fauville et al., 2021; Li and Liu, 2025; Madon and Krishna, 2018; Marsh et al., 2022). Among these challenges, digital fatigue, characterized by mental exhaustion resulting from excessive use of digital devices and platforms, has emerged as a growing concern for both employees and organizations (Reeves et al., 2021; Zalewska-Turzyńska, 2022). Employees are now frequently overwhelmed by the sheer volume of emails, virtual meetings, and the need to remain constantly connected (Sonnentag, 2018). This persistent interaction with digital technologies can contribute to burnout, reduced concentration, and decreased motivation (Fauville et al., 2021; Tarafdar et al., 2017). Thus, understanding the implications of digital fatigue on employee well-being and work outcomes is critically important, particularly in terms of its influence on work engagement—a positive, fulfilling work-related state of mind characterized by vigor, dedication, and absorption (Bakker et al., 2014; Knight et al., 2017).

Building upon this emerging concern, recent studies have begun to explore the antecedents, processes, and consequences of digital fatigue (Gregersen et al., 2023; Hwang et al., 2020; Lee et al., 2021; Reeves et al., 2021). Although prior research has increasingly examined the detrimental impacts of digital fatigue on employees’ psychological well-being, work engagement, and performance (Dixit et al., 2023; Jain et al., 2025; Supriyadi et al., 2025), relatively limited attention has been devoted to understanding under what conditions these negative effects can be mitigated. In particular, while some studies have acknowledged the potential role of contextual resources (Jain et al., 2025), their moderating effects on the digital fatigue–work engagement relationship remain insufficiently explored and conceptually fragmented. Given the complexity of contemporary digital work environments—characterized by persistent connectivity demands and the interplay of internal and external relationships—it is crucial to adopt a more nuanced, contingency-based perspective.

Leadership style represents one such critical contingency. On one hand, transformational leadership, with its emphasis on vision, inspiration, and individualized consideration (Monje-Amor et al., 2020), can potentially buffer the adverse effects of digital fatigue by fostering a supportive atmosphere that energizes employees and redirects their attention from the strains of digital overload to broader collective goals (Le and Lei, 2019; Ling et al., 2008; Monje-Amor et al., 2020). On the other hand, transactional leadership—focused on clear expectations, rewards, and penalties (Jensen et al., 2016; MacKenzie et al., 2001; Pieterse et al., 2009)—may not offer the same emotional scaffolding. While it ensures structured guidance and performance accountability, it may fail to alleviate feelings of fatigue caused by incessant digital demands. As such, differing leadership styles could shape how employees interpret and respond to digital fatigue, and consequently, how it translates into variations in work engagement.

In addition to leadership style, employees’ network ties within and beyond the organization can also play a moderating role (Mitchell et al., 2001; Porter et al., 2022). Internal ties, such as connections with colleagues and supervisors, may provide social support and resource exchange that help employees cope more effectively with digital fatigue (Porter et al., 2019; Tews et al., 2019). Such ties might alleviate feelings of isolation and offer emotional reassurance, thereby fostering sustained engagement. External ties—relationships with clients, partners, or professional communities outside the firm—could likewise supply valuable knowledge, career development opportunities, and broader perspectives (Lechner and Dowling, 2003; Porter et al., 2022). At the same time, these external connections may introduce additional digital demands (e.g., responding to external stakeholders across multiple time zones and platforms), thus complicating employees’ experiences of fatigue. Examining both internal and external network ties is therefore crucial to understanding the nuanced conditions under which digital fatigue influences employee engagement.

Against this backdrop, this study adopts an integrative theoretical perspective combining the COR theory (Hobfoll, 1989) and the JD-R model (Bakker and Demerouti, 2007) to develop a comprehensive framework explaining how digital fatigue affects employees’ work engagement and under what conditions this effect varies. According to COR theory, individuals strive to acquire, maintain, and protect valuable resources, and stress arises when these resources are threatened or depleted (Hobfoll, 1989). From this lens, digital fatigue reflects a state of resource loss that can diminish employees’ energy and motivation, ultimately lowering their engagement levels. In contrast, the JD-R framework emphasizes that employees’ well-being is shaped by the dynamic interplay between job demands and job resources (Bakker and Demerouti, 2007). Drawing on this model, the present study argues that contextual resources—specifically leadership style and network ties—can buffer the negative effects of digital fatigue by replenishing depleted resources or facilitating adaptive coping.

To empirically validate the proposed theoretical framework, this study used survey data collected from 339 employees in Chinese technology firms. By developing and empirically validating this model, our research makes three key theoretical contributions. First, it integrates the COR theory and the JD-R framework to develop a unified resource-based perspective on how digital fatigue influences work engagement, thereby extending the explanatory power of both theories within the context of digital fatigue research. Second, it contributes to leadership research by revealing how transformational and transactional leadership differentially shape the digital fatigue–engagement relationship, identifying leadership style as a critical contextual mechanism that can either buffer or intensify technology-induced strain. Third, it advances organizational social network research by distinguishing the functional roles of internal and external network ties, demonstrating that only internal ties provide immediate socio-emotional support that mitigates digital fatigue, whereas the influence of external ties appears more complex and context-dependent. Furthermore, the managerial implications of this study are noteworthy, as they suggest that managers can mitigate the negative impact of digital fatigue on work engagement by adjusting leadership styles and strengthening specific network ties.

## 2 Theoretical background

### 2.1 Digital fatigue in contemporary work environments

Digital fatigue, broadly defined as a state of mental exhaustion stemming from prolonged and excessive use of digital technologies, has become an increasingly salient issue in modern organizational settings (Dixit et al., 2023; Reeves et al., 2021; Zalewska-Turzyńska, 2022). Although digital fatigue shares surface similarities with traditional work-related fatigue (both reflect energy depletion), it is etiologically and phenomenologically distinct. Traditional fatigue is typically induced by prolonged physical or cognitive effort within bounded work episodes and is well described by mental-fatigue research as a psychobiological state following sustained demanding activity that diminishes cognitive efficiency and motivation (Boksem and Tops, 2008; Pageaux, 2014).

By contrast, digital fatigue emerges from technology-mediated work characterized by persistent connectivity, information/ communication overload, and boundaryless temporal–spatial demands (e.g., continuous notifications, back-to-back videoconferences, pressure for instantaneous replies) (Hwang et al., 2020; Lee et al., 2021), which produce sensory overload, attentional fragmentation, and socio-emotional drain (Tarafdar et al., 2017). Empirically, videoconference/ digital-fatigue research documents multidimensionality—including visual, emotional, social, and motivational facets—rather than a unitary tiredness construct typical of classical fatigue accounts (Fauville et al., 2021; Gregersen et al., 2023). Moreover, ubiquitous ICTs amplify “always-on” demands and cognitive load, thereby differentiating digital fatigue from traditional fatigue not merely in degree but in kind—as a distinct, technology-induced form of resource depletion that constrains opportunities for psychological recovery (Barber and Santuzzi, 2015; Bondanini et al., 2020).

Existing research has illuminated the detrimental effects of digital fatigue on a variety of employee outcomes. For example, elevated levels of digital fatigue are associated with reduced well-being, heightened stress, and impaired cognitive functioning (Reeves et al., 2021; Romero-Rodríguez et al., 2023). Employees experiencing digital fatigue frequently report diminished concentration and decreased motivation, factors that can erode job satisfaction and performance quality over time (Di Leo, 2016; Tarafdar et al., 2017). Moreover, persistent digital fatigue has been linked to withdrawal behaviors, as employees attempt to cope by disengaging from digital interactions and potentially reducing their involvement in collaborative organizational activities (Dixit et al., 2023). Prior studies have also identified several moderating factors that shape the relationship between digital fatigue and employee outcomes, focusing primarily on digital autonomy, team digital norms, psychological safety (Jain et al., 2025), camera or self-view settings (Basch et al., 2025), meeting characteristics (e.g., duration, group size) (Nesher Shoshan and Wehrt, 2021), appearance-related concerns (Ratan et al., 2022), and personality traits (Tan et al., 2022). While these findings provide valuable insights, research on broader contextual moderators remains relatively limited. In particular, interpersonal resources (e.g., leadership style) and structural resources (e.g., internal vs. external network ties) have received less attention—an area this study seeks to advance.

### 2.2 Work engagement

Work engagement refers to a positive, fulfilling, and work-related psychological state characterized by vigor, dedication, and absorption (Bakker and Demerouti, 2008; Schaufeli and Bakker, 2010). Unlike job satisfaction, which primarily reflects an affective response to work conditions (Locke, 1969), work engagement captures an individual’s active investment in their work tasks, encompassing emotional, cognitive, and physical energy (Bakker and Demerouti, 2008).

As a central construct in positive organizational psychology, work engagement represents a self-driven motivational state that enables employees to sustain energy, focus, and persistence even under demanding conditions (Bakker and Demerouti, 2017; Poku et al., 2025; Schaufeli, 2018). Such heightened activation enhances individual outcomes—including well-being, commitment, creativity, and task performance (Christian et al., 2011; Hui et al., 2020; Kim et al., 2013; Radic et al., 2020)—and extends to the collective level by fostering collaboration, innovation, and customer-oriented behavior (Christian et al., 2011; De Spiegelaere et al., 2014; Kim et al., 2017; Knight et al., 2017). From an organizational perspective, engagement functions as a vital mechanism linking leadership behaviors and human resource practices to higher productivity, stronger employee retention, improved organizational performance, and greater customer satisfaction (Khwaja and Yang, 2022; Knight et al., 2017; Kundu and Lata, 2017; Vogel et al., 2022). Consequently, fostering and protecting employee engagement has become a strategic priority for organizations navigating increasingly digital and dynamic work environments.

Given its critical role in enhancing both individual and organizational outcomes, understanding the factors that foster and sustain work engagement has become a central focus of recent research. Previous studies have examined various antecedents of work engagement, ranging from individual traits to organizational factors (Lesener et al., 2019). Individual-level determinants include self-efficacy (Federici and Skaalvik, 2011), resilience (Wang et al., 2011), psychological capital (Tian et al., 2023), personality (Herr et al., 2021), Cultural intelligence (Afsar et al., 2020), and emotional intelligence (Alotaibi et al., 2020). Organizational-level factors, such as leadership behaviors (Islam et al., 2024), organizational support (Aldabbas et al., 2021), learning organization (Malik and Garg, 2020), financial bonuses (Kulikowski and Sedlak, 2020), and organizational climate (Rožman and Štrukelj, 2020), have also been identified as significant contributors. Moreover, contextual variables like corporate culture (Wen et al., 2023), workplace well-being (Baquero, 2023), and work resources (Ng and Tay, 2010) have been shown to enhance engagement.

Despite the rich literature on the antecedents of work engagement, new challenges in the contemporary work environment, such as digital fatigue, warrant further investigation. As remote and hybrid work intensifies, employees experience constant connectivity and digital interruptions that drain attention, heighten cognitive strain, and trigger digital fatigue. Building on prior research, this study seeks to extend existing understanding by examining how digital fatigue interacts with key contextual resources—namely leadership style and network ties—to influence work engagement in modern organizational contexts.

### 2.3 JD-R theory

The JD-R Theory, proposed by Bakker and Demerouti (2007), serves as a foundational framework for understanding how various job characteristics influence employee well-being and performance (Bakker et al., 2023a; Mazzetti et al., 2023; Tang et al., 2024). The JD-R model categorizes job attributes into two broad categories: job demands and job resources (Bakker and Demerouti, 2007; Schaufeli, 2017). Job demands refer to the physical, psychological, social, or organizational aspects of a job that require sustained effort and are therefore associated with certain physiological and psychological costs (Bakker and Demerouti, 2007). In the digital era, employees face intensified demands such as information overload, constant connectivity, and pressure for instant responsiveness across multiple digital platforms (Tarafdar et al., 2017). Digital fatigue represents a strain outcome arising from prolonged exposure to excessive digital demands coupled with insufficient job resources. Within the JD-R framework, such an imbalance triggers a process of resource depletion that undermines employees’ motivational energy and hampers their ability to remain engaged at work.

Conversely, job resources are those aspects of the job that help in achieving work goals, reduce job demands, and stimulate personal growth and development (Bakker and Demerouti, 2007). Resources can be physical, psychological, social, or organizational in nature (Bakker and Demerouti, 2007). Within digitalized work settings, resources such as supportive leadership and cohesive network ties may replenish depleted energy, enhance psychological safety, and promote resilience (Bakker and Demerouti, 2007; Schaufeli, 2017). Accordingly, the JD-R framework in this study is primarily employed to explain the moderating mechanisms through which leadership style and network ties buffer the negative effects of digital fatigue on work engagement.

### 2.4 COR theory

The COR theory, proposed by Hobfoll (1989), offers a comprehensive framework for understanding how individuals respond to stress and resource depletion in demanding environments. Its central premise is that people strive to acquire, maintain, and protect valuable resources—such as time, energy, social support, and self-efficacy—that enable effective functioning. Stress arises when these resources are threatened, lost, or insufficient to meet situational demands, leading to a downward spiral of further depletion (Halbesleben et al., 2014; Hobfoll, 2001).

From this perspective, digital fatigue can be viewed as a manifestation of resource loss caused by continuous exposure to technology-mediated demands, constant connectivity, and cognitive overload. These conditions drain employees’ attentional and emotional resources, impairing their ability to sustain work engagement—a state that fundamentally depends on vigor and motivational energy. Moreover, COR theory emphasizes that resource gain can offset or buffer the effects of resource loss. In this regard, leadership style and network ties serve as contextual resources that may help employees replenish energy, foster psychological safety, and sustain engagement under digital strain.

By adopting the COR framework, this study explains the main effect of digital fatigue on work engagement as a process of resource depletion, while the JD-R framework complements it by illustrating how contextual resources—such as leadership and network ties—can mitigate this negative influence. Integrating COR and JD-R perspectives thus provides a richer understanding of how individuals navigate the challenges of digitalized work environments.

## 3 Hypothesis development

### 3.1 Digital fatigue and work engagement

Drawing on COR theory (Hobfoll, 1989), digital fatigue can be viewed as a state of resource depletion, emerging from continuous exposure to digital communication platforms, multitasking demands, and constant connectivity in technology-mediated work environments (Dixit et al., 2023; Tarafdar et al., 2017). Unlike traditional job stressors—such as workload, role ambiguity or conflict, and interpersonal demands (Levinson et al., 1965; Podsakoff et al., 2007)—digital fatigue imposes a persistent cognitive and emotional burden. Employees must simultaneously manage information overload, frequent online meetings, recurring digital interruptions, and an omnipresent expectation of instant responsiveness (Dixit et al., 2023; Reeves et al., 2021; Zalewska-Turzyńska, 2022). These ongoing demands accelerate the depletion of personal resources, undermining employees’ ability to maintain motivation, attentional focus, and psychological energy at work (Hobfoll et al., 2018).

According to COR theory, the depletion of key resources reduces individuals’ ability to invest in positive work-related states such as work engagement, which is characterized by vigor, dedication, and absorption (Bakker and Demerouti, 2008; Schaufeli and Bakker, 2010). Empirical studies have shown that technology-induced stress and digital overload significantly lower employees’ engagement and well-being by depleting their cognitive and emotional resources (Dixit et al., 2023; Jain et al., 2025; Marsh et al., 2024; Salanova et al., 2013; Supriyadi et al., 2025; Tarafdar et al., 2014). When employees experience ongoing digital fatigue, they may struggle to maintain concentration, lose their sense of accomplishment, and withdraw psychologically from their work (Jain et al., 2025; Supriyadi et al., 2025). Over time, such resource erosion undermines the motivational foundation of engagement, leading to reduced vigor and diminished dedication.

In line with these theoretical and empirical insights, it is reasonable to expect that employees experiencing higher levels of digital fatigue will display lower engagement in their work roles. The continuous depletion of cognitive and emotional resources caused by excessive digital demands constrains employees’ ability to sustain energy and focus, thereby hindering their overall engagement. Thus, the following hypothesis is proposed:

*H1*: Digital fatigue is negatively related to work engagement.

### 3.2 The moderating role of leadership style

Leadership style is a critical contextual factor that shapes how employees interpret, respond to, and manage challenges in their work environment (Bakker et al., 2023b; Marsh et al., 2024). Within the JD-R framework, leadership represents a key social job resource that influences how employees cope with stressors and conserve their psychological energy (Bakker and Demerouti, 2007). In technology-mediated work settings, where digital strain arises from information overload, constant connectivity, and frequent interruptions (Dixit et al., 2023; Zalewska-Turzyńska, 2022), leadership behaviors may significantly affect how employees experience and manage resource depletion (Wang et al., 2011). Depending on the leader’s style and approach, such influence can either alleviate or exacerbate the detrimental effects of digital fatigue on work engagement. Two prominent leadership approaches—transformational and transactional (Burns, 1978)—are particularly relevant for understanding these dynamics.

Transformational leadership refers to a leadership style that motivates and inspires followers by articulating a compelling vision, fostering intellectual stimulation, and providing individualized consideration (Bass et al., 1987; Bono and Judge, 2004; Ling et al., 2008; Seltzer and Bass, 1990). Such leaders emphasize meaning, growth, and shared purpose, thereby elevating employees’ intrinsic motivation and commitment (Bass et al., 1987). Within the JD-R framework, transformational leadership functions as a key motivational resource that enhances employees’ ability to cope with stressors and recover from resource depletion (Bakker and Demerouti, 2017). In digitalized work environments, transformational leaders can help employees reinterpret demanding digital conditions—such as constant connectivity and information overload—as opportunities for development rather than as exhausting burdens (Ling et al., 2008; Seltzer and Bass, 1990). By cultivating a supportive and trusting climate, these leaders provide psychological and emotional resources that replenish employees’ depleted energy and sustain their engagement (Burns, 1978; MacKenzie et al., 2001). Consequently, transformational leadership is expected to buffer the detrimental effect of digital fatigue on work engagement by fostering resource replenishment and maintaining employees’ vigor, dedication, and absorption. Accordingly, the following hypothesis is proposed:

*H2*: Transformational leadership weakens the negative relationship between digital fatigue and work engagement.

In contrast, transactional leadership refers to a leadership style that emphasizes the exchange relationship between leaders and followers through contingent rewards, performance monitoring, and corrective actions (Bono and Judge, 2004; Burns, 1978). Unlike transformational leaders who inspire and empower, transactional leaders primarily focus on clarifying roles, setting performance expectations, and ensuring compliance through structured control mechanisms (Abbas and Ali, 2023). Within the JD-R framework, such behaviors provide instrumental resources—such as task clarity and feedback—but offer limited motivational or psychological resources (Bakker and Demerouti, 2017).

In digitalized work environments, where employees already face high information load and constant connectivity (Barber and Santuzzi, 2015; Marsh et al., 2022), transactional leadership may unintentionally intensify digital strain by reinforcing external pressures for responsiveness and productivity. Although this style enhances consistency and short-term efficiency (Abbas and Ali, 2023), its emphasis on control and extrinsic motivation often fails to provide sufficient emotional resources and autonomy needed for recovery from digital fatigue (Abbas and Ali, 2023; Bono and Judge, 2004). Consequently, employees may perceive digital fatigue as an inescapable burden that must be managed independently—accelerating cognitive and emotional depletion over time and ultimately weakening their vigor, dedication, and absorption at work. Accordingly, the following hypothesis is proposed:

*H3*: Transactional leadership strengthens the negative relationship between digital fatigue and work engagement.

### 3.3 The moderating role of employee’s network ties

In addition to leadership styles, employees’ network ties—both within and beyond organizational boundaries—constitute critical social resources that shape their responses to digital fatigue (Porter et al., 2022). Within the JD-R framework, such ties function as social job resources that can replenish psychological energy, facilitate information exchange, and buffer stress arising from digital overload (Bakker and Demerouti, 2007). The strength and nature of these ties determine whether employees can access supportive resources or instead encounter additional demands in technology-mediated work environments.

Internal network ties, consisting of collegial relationships and connections with supervisors and peers within the firm, can serve as vital psychosocial resources (Mitchell et al., 2001). Such ties enable employees to share coping strategies, exchange pertinent information, and provide emotional reassurance (Leana and van Buren, 1999; Mitchell et al., 2001; Porter et al., 2019). When employees are confronted with overwhelming digital demands, robust internal ties offer a sense of solidarity and mutual understanding, thereby alleviating feelings of isolation or helplessness (Tews et al., 2019). By facilitating resource-rich interactions that counterbalance cognitive strain and emotional depletion, internal ties can mitigate the detrimental effects of digital fatigue on employees’ willingness and ability to remain engaged in their work. Therefore, we hypothesize that:

*H4*: Employees’ internal network ties weaken the negative relationship between digital fatigue and work engagement.

In contrast, external network ties, encompassing relationships with clients, business partners, and professional communities beyond the firm’s boundaries (Houghton et al., 2009), may intensify the link between digital fatigue and reduced engagement. While these ties can provide valuable insights, career opportunities, and industry knowledge (Lechner and Dowling, 2003; Porter et al., 2022), they often demand sustained digital interactions across multiple platforms and time zones. This sustained connectivity imposes additional cognitive and emotional demands, increasing the sense of digital overload (Barley et al., 2011; Mazmanian et al., 2013). Moreover, without the inherent trust, empathy, and shared organizational context often found in internal networks, external ties may offer less emotional support and more digitally driven expectations (Labianca, 2004; Leana and van Buren, 1999; Nahapiet and Ghoshal, 1998). Consequently, these external connections can exacerbate digital fatigue and its negative influence on employees’ capacity to remain fully engaged. Accordingly, we propose the following hypothesis:

*H5*: Employees’ external network ties strengthen the negative relationship between digital fatigue and work engagement.

**Figure 1:**
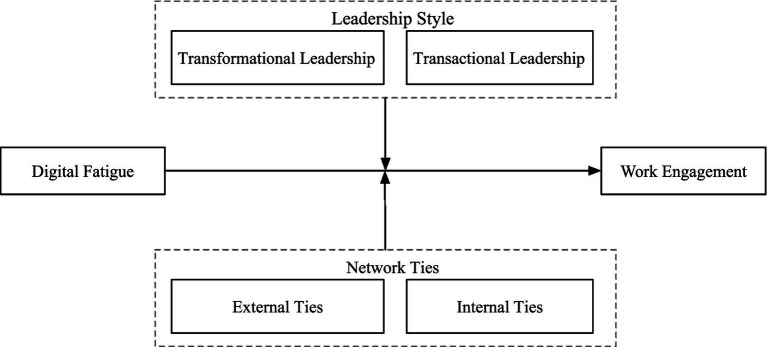
Theoretical framework.

Figure 1 illustrates our conceptual model that summarises all the proposed relationships.

## 4 Method

### 4.1 Sample and data collection

To test our hypotheses, we collaborated with a professional survey company to collect data from full-time employees working in technology firms. These organizations, which rely extensively on digital tools and platforms, provide a highly relevant context for examining issues related to digital fatigue. To ensure that participants had sufficient exposure to digital work demands, the sample was restricted to general employees and frontline managers.

The survey company was commissioned to collect 350 questionnaires. A total of 646 responses were initially received. After the survey company’s preliminary screening, 291 responses were excluded because (1) respondents were neither frontline managers nor general employees, (2) respondents’ job roles did not involve extensive use of digital tools, (3) completion times were abnormally short or excessively long, (4) the employing organizations did not meet the inclusion criteria (i.e., non-technology-oriented firms) and (5) the questionnaires were incomplete. This yielded 355 usable responses, corresponding to an effective response rate of approximately 54.95%. Subsequently, we conducted an additional manual verification of the remaining questionnaires to further ensure data quality. This process involved checking for (1) failure to pass attention-check items, (2) straight-line or otherwise repetitive response patterns, and (3) logically inconsistent answers. As a result, 16 additional invalid responses were removed, producing a final sample of 339 valid questionnaires and an effective response rate of 95.49%.

Among the respondents, 72.3% were male and 27.7% were female. The majority of participants (62.8%) were aged between 26 and 35, followed by 20.1% aged 25 or below, 15.9% aged between 36 and 45, and 1.2% aged 46 or above. Regarding educational background, most respondents (79.9%) held a bachelor’s degree, while 11.8% had an associate degree, and 8.3% had a master’s degree. In terms of job positions, 19.5% of the respondents were frontline managers, while the remaining 80.5% were general employees.

The organizations represented in the sample demonstrated diverse characteristics. Regarding the length of establishment, 24.8% of the companies had been operating for 3–5 years, 22.7% for 5–10 years, 17.1% for 1–3 years, 15.0% for over 10 years, and 1.5% for less than 1 year. In terms of company size, 47.2% of the firms employed 101–200 people, 17.7% employed 51–100 people, 12.4% employed 201–300 people, 13.3% employed fewer than 50 people, and 9.4% employed more than 301 people. Regarding ownership structure, the majority of firms were privately owned (81.7%), followed by 10.3% that were state-owned, 2.9% that were foreign-funded, 2.9% that were collectively owned, and 2.1% that were joint ventures.

### 4.2 Measurement

The measurement scales used in this study were sourced from reputable scholarly research. To ensure their suitability, the English-language scales underwent a rigorous translation and back-translation procedure (Brislin, 1980), supplemented by contextual and cultural adjustments tailored to the unique Chinese work environment. All items were rated on a five-point Likert scale and were pretested prior to the main survey.

#### 4.2.1 Digital fatigue

Drawing upon prior research on digital work environments and employee fatigue (Di Leo, 2016; Dixit et al., 2023; Fauville et al., 2021; Reeves et al., 2021; Zalewska-Turzyńska, 2022), this study conceptualizes digital fatigue as a multifaceted construct encompassing visual, emotional, social, and motivational dimensions (Fauville et al., 2021). These dimensions collectively capture the complex nature of fatigue arising from prolonged and intensive engagement with digital technologies. The scale developed by Fauville et al. (2021) was adapted for this study, comprising ten items, such as “*Extended engagement with digital technologies leaves me feeling emotionally drained*.” The construct showed high internal consistency (Cronbach’s *α* = 0.919; CR = 0.933).

#### 4.2.2 Work engagement

*Work engagement* was assessed using the nine-item short version of the Utrecht Work Engagement Scale (UWES-9; Schaufeli et al., 2006), covering vigor, dedication, and absorption. A sample item is “*At my job, I feel bursting with energy.*” The scale showed excellent reliability (Cronbach’s α = 0.938; CR = 0.948).

#### 4.2.3 Leadership style

*Leadership style* was measured using Bass and Avolio’s (1995) Multifactor Leadership Questionnaire (MLQ). Transformational leadership was measured with eight items (e.g., “*He/She encourages employees to consider different viewpoints when analyzing problems*”) (Cronbach’s α = 0.938; CR = 0.949), while transactional leadership was measured with five items (e.g., “*He/She makes it clear to employees what rewards they will receive for achieving goals*”) (Cronbach’s α = 0.948; CR = 0.960).

#### 4.2.4 Network ties

To evaluate employees’ internal and external network ties, we applied the ego-centric name generator method (Marsden, 1990). Following Porter et al. (2022), participants were asked to list five close colleagues within their organization. If fewer than five close colleagues were available, participants listed additional colleagues with whom they frequently interacted. They then rated their closeness to these five individuals on a five-point Likert scale (1 = “Not at all close,” 5 = “Very close”). The average rating of these five colleagues indicated the strength of the employee’s internal network ties. Similarly, participants identified five friends outside their firm (and not family members) with whom they regularly discussed work and life matters. If they had fewer than five such friends, participants listed additional acquaintances with whom they had meaningful interactions. Closeness to these external network members was rated using the same five-point scale. The average closeness rating of these five friends indicated the strength of the employee’s external network ties. Both measures demonstrated satisfactory reliability (Cronbach’s α > 0.84; CR > 0.85).

Additionally, demographic and organizational factors potentially influencing work engagement were controlled for, including age, gender, educational level, job position, firm ownership, firm size, and founding time (Aggarwal et al., 2020; Blaique et al., 2022; Monje-Amor et al., 2021; Yan et al., 2021). For details of all measurement items, factor loadings, and AVE values, please refer to Appendix A.

## 5 Analyses and results

### 5.1 Measurement model

To comprehensively evaluate the constructs in this study, a series of analytical procedures were conducted. As a preliminary step, an exploratory factor analysis (EFA) was performed. The results confirmed that the data were suitable for factor analysis (KMO = 0.927; Bartlett’s test of sphericity: *χ*^2^ = 10413.725, df = 861, *p* < 0.001). Six factors were extracted, consistent with the theoretical constructs, and all items loaded strongly on their intended factors with minimal cross-loadings. The detailed rotated component matrix is reported in Appendix B.

Subsequently, a confirmatory factor analysis (CFA) was conducted to further assess the measurement model. The hypothesized six-factor model demonstrated the best fit to the data compared with alternative models, including five-factor, four-factor, three-factor, two-factor, one-factor, and seven-factor structures. Specifically, the six-factor model showed satisfactory fit indices (CMIN/DF = 1.452 < 3; RMSEA = 0.037 < 0.05; SRMR = 0.0414 < 0.05; CFI = 0.964; TLI = 0.961; IFI = 0.964), all of which surpassed conventional thresholds. By contrast, the alternative models exhibited significantly poorer fit (see Table 1). Moreover, all items in the six-factor model demonstrated significant standardized loadings on their respective constructs (*p* < 0.001), ranging from 0.733 to 0.925, providing robust evidence of construct validity.

**Table 1:** Comparison of alternative measurement models.

Model	CMIN/DF	RMSEA	SRMR	CFI	TLI	IFI
Six-factor model (hypothesized)	1.452	0.037	0.0414	0.964	0.961	0.964
Five-factor model^a^	2.201	0.060	0.0678	0.904	0.879	0.905
Four-factor model^b^	3.928	0.093	0.0957	0.765	0.749	0.767
Three-factor model^c^	5.708	0.118	0.1367	0.620	0.596	0.622
Two-factor model^d^	6.147	0.123	0.1393	0.583	0.558	0.584
One-factor model^e^	7.227	0.136	0.1562	0.492	0.463	0.494
Seven-factor model^f^	2.136	0.058	0.0457	0.910	0.902	0.910

a Internal and external ties combined.

b Transformational and transactional leadership combined.

c Work engagement and digital fatigue further merged.

d All variables grouped into two broad factors (ties + fatigue + engagement vs. leadership).

e All items constrained to a single factor.

f Digital fatigue split into two dimensions.

Internal consistency was satisfactory, with Cronbach’s alpha values ranging from 0.842 to 0.948 and composite reliability (CR) values ranging from 0.845 to 0.960, all above the recommended threshold of 0.70 (Hair et al., 2014; Santos, 1999). Convergent validity was supported, as both factor loadings and average variance extracted (AVE) values exceeded the 0.50 benchmark (Hair et al., 2014). A detailed summary of these results is provided in Appendix A.

Discriminant validity was further confirmed using Fornell and Larcker’s (1981) criterion: the square root of each construct’s AVE (reported on the diagonal of Table 2) exceeded the correlations between constructs. This demonstrates that the constructs are conceptually distinct and not excessively interrelated.

**Table 2:** Means, standard deviation and correlations.

Variable	Mean	S.D.	1	2	3	4	5	6	7	8	9	10	11	12	13
1. Age	1.982	0.639	**-**												
2. Gender	1.277	0.448	0.028	–											
3. Educational level	2.965	0.447	−0.085	0.005	–										
4. Job position	3.805	0.397	−0.06	−0.062	−0.039	–									
5. Firm ownership	2.047	0.66	0.037	−0.024	−0.024	0.035	–								
6. Firm size	2.87	1.094	−0.101	0.019	−0.118*	0.16**	−0.041	–							
7. Founding time	3.705	1.149	−0.011	0.033	−0.164**	0.075	−0.013	0.403***	–						
8. Digital fatigue	3.767	0.843	0.046	0.053	0.047	−0.123*	0.122*	−0.251***	−0.033	**0.762**					
9. Work engagement	3.516	0.962	0.026	−0.112*	−0.078	0.071	0.076	0.134*	0.071	−0.356***	**0.819**				
10. Transformational	3.509	1.069	0.07	−0.137*	−0.119*	0.009	0.086	0.05	0.027	−0.213***	0.648***	**0.836**			
11. Transactional	3.031	1.342	−0.119*	0.123*	−0.048	−0.001	−0.052	0.265***	0.182**	−0.043	−0.307***	−0.409***	**0.910**		
12. Internal ties	3.287	0.976	0.09	−0.103	−0.024	0.033	0.084	−0.051	−0.126*	−0.025	0.578***	0.552***	−0.425***	**0.787**	
13. External ties	4.127	0.757	−0.049	0.004	−0.006	−0.028	−0.08	−0.093	−0.014	−0.002	−0.044	−0.072	−0.024	−0.052	**0.783**

1. *N* = 339; 2. *, **, and *** denote significance at the 0.05, 0.01, and 0.001 levels, respectively; 3. The square roots of the AVE values are reported on the diagonal in bold, while the inter-construct correlations are shown below the diagonal; 4. Control variables were coded as follows: gender (1 = male, 2 = female), age (1 = ≤25 years, 2 = 26–35 years, 3 = 36–45 years, 4 = ≥46 years), educational level (1 = high school or below, 2 = junior college, 3 = bachelor’s degree, 4 = master’s degree or above), job position (1 = chairman/general manager, 2 = department manager/supervisor, 3 = frontline manager, 4 = general employee), firm founding time (1 = ≤1 year, 2 = 1–3 years, 3 = 3–5 years, 4 = 5–10 years, 5 = ≥10 years), firm size (1 = ≤50 employees, 2 = 51–100, 3 = 101–200, 4 = 201–300, 5 = ≥301), and ownership type (1 = state-owned, 2 = privately owned, 3 = joint venture, 4 = foreign-funded, 5 = collectively owned).

Finally, to evaluate the potential influence of nonresponse bias, the sample was divided into two groups—early and late respondents—based on their questionnaire submission dates. A t-test analysis was performed to compare the founding time and organizational size between these groups. The results revealed no statistically significant differences in founding time (*p* = 0.375) or organizational size (*p* = 0.196), indicating that nonresponse bias did not substantially affect the study’s findings (Armstrong and Overton, 1977).

Taken together, these analyses provide strong evidence of the measurement model’s reliability, convergent and discriminant validity, and overall adequacy. This solid measurement foundation supports the validity of the subsequent hypothesis testing (Garver and Mentzer, 1999; Hair et al., 2014).

### 5.2 Common method bias

Given the reliance on self-reported data in this research, addressing potential common method bias (CMB) is essential (Podsakoff and Organ, 1986). To assess this issue, *Harman’s single-factor test* was employed by conducting an unrotated principal component analysis, including all variables with eigenvalues greater than 1. The results showed that the first factor explained 30.285% of the total variance, which is well below the 50% threshold. These findings suggest that the impact of CMB in this study is minimal (Hair et al., 2014).

To further assess CMB, a single unmeasured latent method factor approach was applied (Podsakoff et al., 2003). This involved incorporating a first-order factor, loaded with all observed variables, into the theoretical model. The fit indices for this model were as follows: CMIN/DF = 1.396(< 3), RMSEA = 0.0355 (< 0.05), SRMR = 0.0414 (< 0.05), CFI = 0.970, TLI = 0.966, and IFI = 0.971. Notably, the inclusion of the method factor did not enhance the goodness-of-fit compared to the original six-factor model, providing further evidence that CMB is not a significant concern in this study.

### 5.3 Hypothesis testing

Table 3 presents the results of the hierarchical regression analyses conducted to test the study’s hypotheses. Model 1 incorporates only the control variables, while Models 2 through 4 sequentially introduce the main effects and moderating variables. Variance inflation factors (VIFs) across all models remain below the commonly accepted threshold of 10, indicating that multicollinearity is not a concern (Hair et al., 2014).

**Table 3:** Regression analysis results.

Variables	Hypothesis	Work engagement			
		Model 1	Model 2	Model 3	Model 4
Control variable					
Age		0.037	0.041	−0.008	−0.007
Gender		−0.112^*^	−0.093	−0.027	−0.038
Educational level		−0.054	−0.043	0.001^*^	−0.025
Job position		0.041	0.010	0.021	−0.022
Firm ownership		0.074	0.116^*^	0.041	0.043
Firm size		0.124^*^	0.031	0.035	−0.019
Founding time		0.014	0.044	0.037	0.096^*^
Main effect					
Digital Fatigue	*H1*		−0.355^***^	−0.255^***^	−0.297^***^
Moderating effect					
Transformational leadership				0.467^***^	
Transactional leadership				−0.107^*^	
Transformational * Digital Fatigue	*H2*			0.129^*^	
Transactional* Digital Fatigue	*H3*			−0.112^*^	
Internal ties					0.499^***^
External ties					−0.013
Internal ties* Digital Fatigue	*H4*				0.230^***^
External ties* Digital Fatigue	*H5*				−0.040
Max *VIF*		1.237	1.313	1.705	1.356
*R* ^2^		0.044	0.158	0.522	0.532
Adjusted *R*^2^		0.024	0.138	0.504	0.505
*F*		2.166^*^	7.748^***^	29.629^***^	29.745^***^

*n* = 339; *, **, *** are signicant at the 0.05, 0.01 and 0.00 levels, respectively; VIF refers to the variance inflation factor.

The results of Model 2 reveal that digital fatigue has a significant negative impact on work engagement (*β* = −0.355, *t* = −6.695, *p* < 0.001). Compared to Model 1, both *R*^2^ and adjusted *R*^2^ values show substantial increases, providing support for *Hypothesis 1.* Regarding the contingent effects of leadership style, Model 3 indicates that the interaction between transformational leadership and digital fatigue significantly influences work engagement (*β* = 0.129, *t* = 2.573, *p* < 0.05). This finding suggests that transformational leadership buffers the negative effect of digital fatigue on work engagement, thus supporting *Hypothesis 2*. Moreover, Model 3 shows that the interaction between transactional leadership and digital fatigue is also significant (*β* = −0.112, *t* = −2.501, *p* < 0.05), suggesting that transactional leadership amplifies the negative relationship between digital fatigue and work engagement. Therefore, H*ypothesis 3* is confirmed. The notable improvements in *R*^2^ and adjusted *R*^2^ values from Model 2 to Model 3 further validate the moderating role of leadership style.

Turning to employees’ network ties, Model 4 demonstrates that the interaction between internal ties and digital fatigue significantly impacts work engagement (*β* = 0.230, *t* = 5.172, *p* < 0.001). This result indicates that strong internal network ties mitigate the adverse effects of digital fatigue on work engagement, supporting *Hypothesis 4.* However, the interaction between external ties and digital fatigue does not achieve statistical significance (*β* = −0.040, *t* = −0.993, *p* > 0.05). This finding suggests that external network ties do not moderate the relationship between digital fatigue and work engagement, leading to the rejection of *Hypothesis 5*.

**Figure 2:**
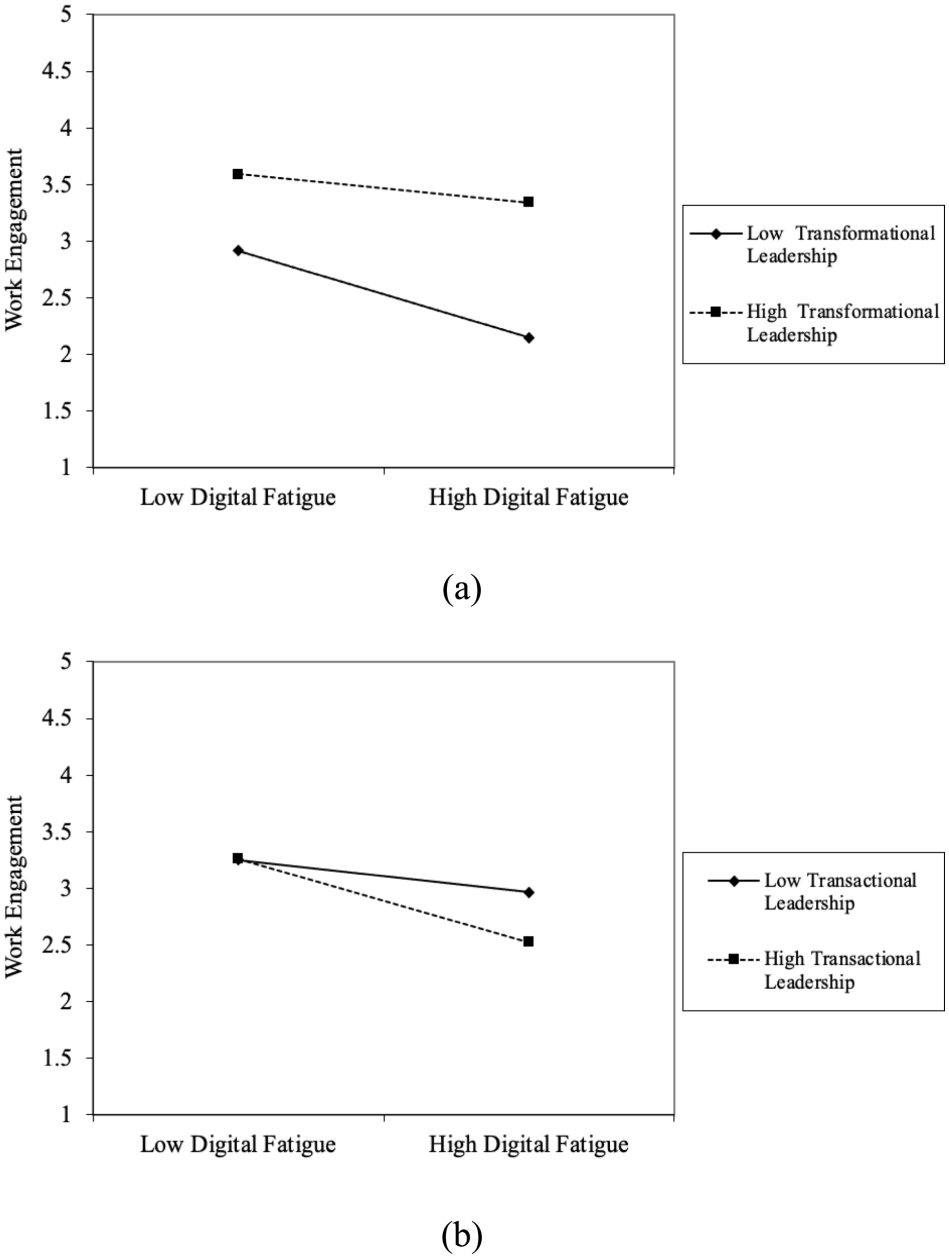
The contingent effect of leadership style: **(a)** The moderating effect of transformational leadership on the relationship between digital fatigue and work engagement. **(b)** The moderating effect of transactional leadership on the relationship between digital fatigue and work engagement.

**Figure 3:**
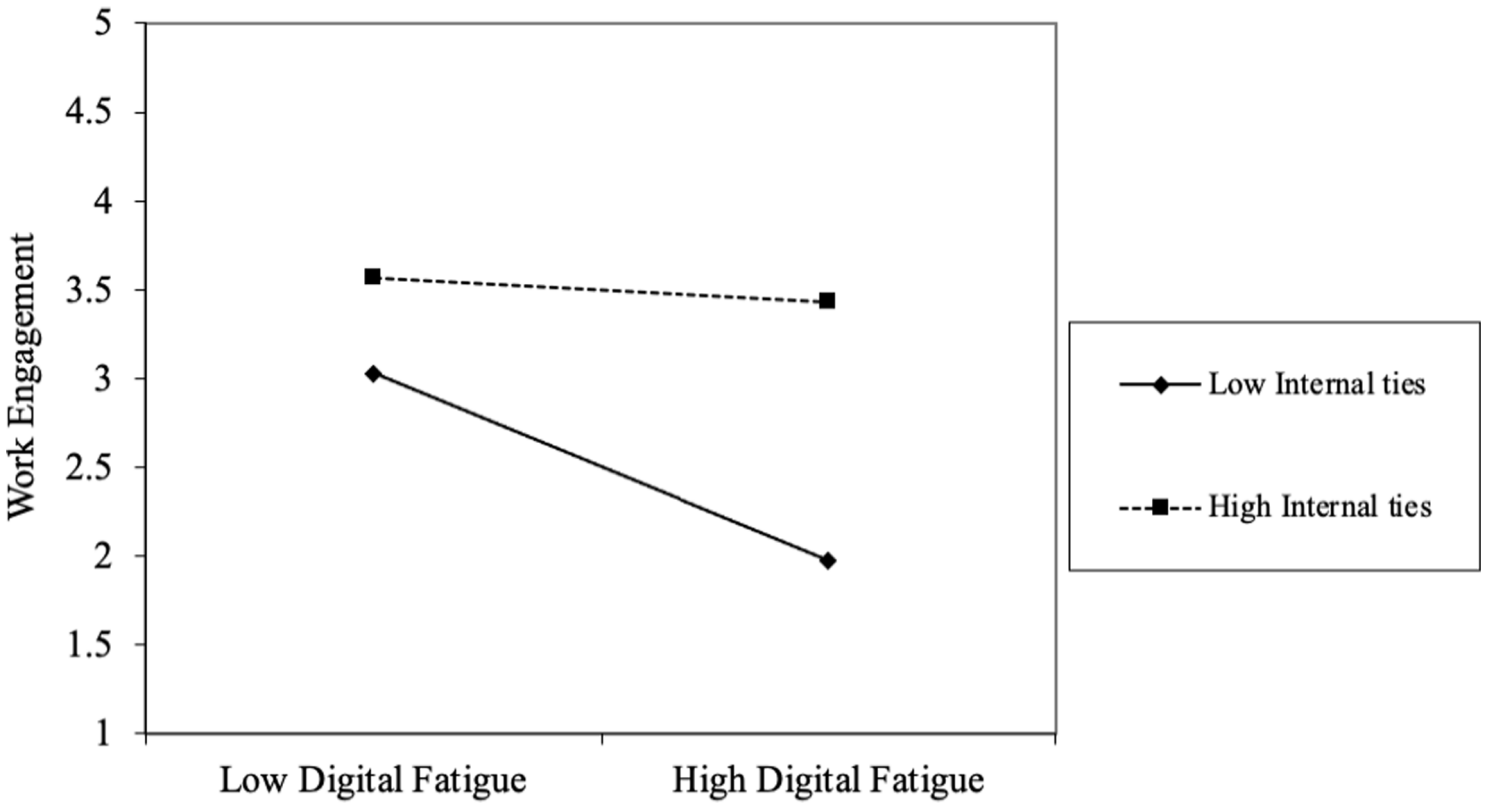
The contingent effect of employees’ network ties.

For a more illustrative depiction of the contingent effects, this study used the method proposed by Preacher et al. (2006) for computing simple slopes at varying levels of the moderator. Figures 2, 3 present the plotted relationships for transformational leadership, transactional leadership, and internal ties, respectively.

As shown in Figure 2a, which examines the moderating effect of transformational leadership, the negative slope of the relationship between digital fatigue and work engagement is less steep under conditions of higher transformational leadership. When digital fatigue is low, employees under both low and high transformational leadership conditions report relatively high engagement. However, as digital fatigue intensifies, employees experiencing high transformational leadership show a more moderate decline in engagement. This pattern supports the notion that transformational leadership acts as a buffer, weakening the adverse impact of digital fatigue on work engagement.

Figure 2b, depicting the moderating role of transactional leadership, reveals a different trend. Although work engagement decreases as digital fatigue rises for both groups, the decline is more pronounced for employees with higher transactional leadership. When digital fatigue is low, differences in engagement between low and high transactional leadership conditions are relatively minor. Yet as digital fatigue increases, employees in the high transactional leadership condition experience a sharper drop in engagement. This outcome aligns with the hypothesis that transactional leadership amplifies the negative influence of digital fatigue on work engagement.

Finally, as illustrated in Figure 3, internal network ties significantly mitigate the detrimental effect of digital fatigue. Although employees with both low and high internal ties show comparable levels of engagement when digital fatigue is minimal, a clear divergence emerges as fatigue intensifies. Under high levels of digital fatigue, employees with stronger internal ties maintain substantially higher work engagement than those with weaker ties. This finding highlights the protective role of internal social resources, demonstrating that internal network ties can help sustain employee engagement even in demanding digital work environments.

## 6 Discussion

### 6.1 Findings

This study aimed to clarify the relationship between digital fatigue and work engagement, as well as to identify key contingency factors that shape this relationship within digitally intensive work environments. Drawing on the JD-R theory (Bakker and Demerouti, 2007) and COR theory (Hobfoll, 1989), the findings provide empirical evidence that digital fatigue negatively affects employees’ engagement levels, which aligns with prior research showing that technology-related strain diminishes employees’ well-being, motivation, and engagement (Dixit et al., 2023; Jain et al., 2025; Salanova et al., 2013; Supriyadi et al., 2025; Tarafdar et al., 2017). In other words, the cognitive and emotional depletion incurred by continuous exposure to digital demands—such as incessant communications and rapid-response expectations—significantly reduce employees’ vigor, dedication, and absorption. This result underscores the importance of considering digital fatigue as a critical job demand that can undermine motivational states in contemporary organizational settings.

Beyond establishing this main effect, the study demonstrates that the impact of digital fatigue on work engagement is contingent upon leadership style. Transformational leadership emerges as a protective factor, consistent with evidence that inspirational and supportive leadership behaviors enhance employees’ resilience and intrinsic motivation (Bakker et al., 2023b; Monje-Amor et al., 2020). Under leaders who inspire, support, and foster an environment receptive to employee needs (Pieterse et al., 2009), the detrimental effect of digital fatigue is notably weaker. Employees experiencing both high digital fatigue and strong transformational leadership are better able to maintain their engagement, likely due to the emotional and psychological resources that such leadership provides (Bakker et al., 2023b). Conversely, transactional leadership, with its emphasis on goal attainment, monitoring, and rewards contingent on compliance (Pieterse et al., 2009), exacerbates the negative relationship. In conditions characterized by substantial digital fatigue, transactional leadership intensifies feelings of strain and reduces engagement, indicating that employees lacking relational and motivational support may find digital overload more burdensome. This finding is consistent with prior research showing that transactional leadership tends to amplify stress and undermine engagement under high job demands (Lyons and Schneider, 2009).

In addition to leadership style, the study highlights the role of internal network ties. Prior research shows that supportive internal networks can enhance employee outcomes such as work attitudes and effectiveness (Chiaburu and Harrison, 2008). Consistent with these findings, our findings indicate that employees embedded in supportive internal networks—characterized by trust, emotional support, and resource sharing—are better equipped to cope with digital fatigue and sustain higher levels of engagement. Colleagues and supervisors who understand and share one’s work context can offer strategies, empathy, and reassurance, thus mitigating the strain that stems from digital demands. However, external network ties do not show a significant moderating effect. Although previous studies suggest that such ties can offer access to valuable information and professional opportunities (Bernardino et al., 2023; Zheng et al., 2019), they may not provide the immediate socio-emotional support required to buffer the draining effects of digital overload. Consequently, these results suggest that not all network connections are equally beneficial for sustaining engagement in the face of digital fatigue.

### 6.2 Theoretical contributions

First, this study integrates the COR theory and the JD-R framework to develop a unified perspective on how digital fatigue influences work engagement. Although prior studies have combined these two theories in broader research on work stress and employee well-being (Bakker et al., 2023c; Xanthopoulou et al., 2007), few have applied their integration to understand the mechanisms through which digital fatigue influences work engagement in digitalized work environments. Building on COR theory, this study explains why digital fatigue undermines engagement through the process of resource depletion (Hobfoll, 1989; Hobfoll et al., 2018). Meanwhile, drawing on the JD-R framework, it clarifies when and under what conditions contextual resources—such as leadership style and network ties—buffer or amplify this depletion (Bakker and Demerouti, 2007). By combining these perspectives, the study advances theoretical understanding from a static view of digital fatigue as a mere strain toward a dynamic resource-balance model, in which resource loss and compensation jointly determine employees’ engagement. This integration enriches existing COR–JD-R research by extending its explanatory power to digitally intensive work settings, where cognitive, emotional, and social resources are persistently challenged by technology-driven demands.

Second, this study contribute to the leadership literature by revealing how distinct leadership styles modulate the digital fatigue–work engagement link. While prior research has established that transformational and transactional leadership differentially affect employee motivation and performance (Bass and Riggio, 2006; Judge and Piccolo, 2004), few studies have examined their roles in technology-mediated work environments where employees experience continuous digital strain. Our findings show that transformational leadership—characterized by inspirational vision, individualized consideration, and intellectual stimulation (Bass et al., 1987; Monje-Amor et al., 2020)—buffers against the depletion caused by digital fatigue, thereby reinforcing employees’ motivational states. In contrast, transactional leadership—anchored in contingent rewards and performance monitoring (Abbas and Ali, 2023; Pieterse et al., 2009)—intensifies the strain of digital fatigue. These patterns sharpen the conceptual contours of leadership theory in digitally intensive contexts, demonstrating that not all leader behaviors are equally efficacious in sustaining engagement amid pervasive technological demands. By bridging leadership and digital fatigue research, this study identifies leadership style as a critical contextual mechanism that can either mitigate or exacerbate technology-induced stress outcomes.

Third, this study advances the organizational social network literature by unpacking the differentiated roles of internal and external network ties in shaping employees’ responses to digital fatigue. Prior research has shown that social ties can provide emotional support, information, and resources that alleviate stress and enhance engagement (Cohen and Wills, 1985; Robertson et al., 2020; Viswesvaran et al., 1999; Wu et al., 2024). At the same time, scholars have also recognized that certain network connections may impose obligations, increase coordination demands, or create information overload, thereby exacerbating strain (Gargiulo and Benassi, 2000; Portes, 1998; Shen et al., 2022). Building on this dual perspective, our findings confirm the beneficial role of internal network ties, which provide trust, shared understanding, and timely socio-emotional support that buffer the adverse effects of digital fatigue on engagement (Mitchell et al., 2001). However, the hypothesized amplifying effect of external ties on the digital fatigue–engagement relationship was not supported. This result suggests that while external ties may increase exposure to cross-boundary interactions and digital demands, their influence may be more complex or contingent than theorized. These findings refine existing social network perspectives by indicating that the functional value of network ties is context-dependent and asymmetric—internal ties serve as immediate social resources, whereas the impact of external ties on employees’ well-being and engagement requires further empirical clarification in digitalized work settings.

### 6.3 Managerial implications

The findings provide actionable guidance for managers and organizations striving to maintain and enhance work engagement in the face of escalating digital demands. First, managers should acknowledge the adverse effects of digital fatigue (Jain et al., 2025; Supriyadi et al., 2025) and implement targeted strategies to reduce digital overload and facilitate resource recovery. In line with the resource depletion logic of COR theory, organizations should focus on restoring employees’ cognitive, emotional, and social resources rather than solely reducing digital exposure (Hobfoll, 1989). This can be achieved by designing work systems that balance technological efficiency with opportunities for recovery—for example, ensuring reasonable workload distribution, allowing autonomy over digital communication pacing, and fostering a supportive climate where employees can voice digital strain without stigma. By addressing the underlying resource imbalance rather than isolated symptoms of fatigue, organizations can better sustain employees’ engagement in technology-mediated work settings.

Second, managers should recognize the pivotal role of transformational leadership behaviors in sustaining employee engagement under digital strain. Leaders who articulate a clear and inspiring vision, show genuine empathy toward employees’ digital challenges, and provide individualized support can help employees reinterpret heavy digital demands as meaningful and achievable goals. Such leaders are encouraged to engage in regular check-ins to identify early signs of digital fatigue, adjust workloads or communication expectations, and model healthy digital behaviors, such as setting boundaries and prioritizing recovery. Managerial training and leadership development programs should therefore focus on strengthening emotional intelligence, motivational communication, and resource-enabling skills, enabling leaders to act as active buffers against employees’ resource depletion. Conversely, leaders relying predominantly on transactional tactics may need to adopt more empathetic and resourceful approaches to prevent further erosion of engagement in digital-intensive contexts.

Third, managers should actively strengthen internal network ties to provide employees with the social and emotional resources needed to cope with digital fatigue. To achieve this, managers can facilitate frequent cross-department collaboration, organize small-group projects, and encourage peer-to-peer mentoring that fosters trust and open communication. Building informal communities—such as virtual coffee chats, employee interest groups, or internal social platforms—can also enhance connectedness and create safe spaces for sharing experiences and coping strategies. By reinforcing these ties, organizations can ensure that employees have the social and emotional support necessary to cope effectively with digital overload.

### 6.4 Limitations and future research directions

Despite its contributions, this study has several limitations that open avenues for future research. First, the data were collected from technology firms in China, which may limit the generalizability of the findings to other cultural or industrial contexts. Digital work cultures, leadership norms, and social network structures vary globally; subsequent research could replicate and extend this study in different settings to enhance external validity. Additionally, the sample comprised firms with diverse ownership structures, including private, state-owned, foreign-funded, collectively owned, and joint-venture enterprises. While this diversity enhances the representativeness of the dataset, it may also introduce contextual heterogeneity. Differences in governance mechanisms, leadership practices, and organizational climates across ownership types could influence the relationships observed in this study. Future research could employ stratified or comparative analyses to examine whether ownership structure moderates the effects of digital fatigue on work engagement.

Second, the cross-sectional research design precludes definitive causal inferences. Although the theoretical framework and robust statistical analysis support the proposed relationships, longitudinal or experimental studies would be more conclusive in establishing temporal order and causality. Future research could track changes in digital fatigue and engagement over time or introduce interventions to strengthen internal ties or alter leadership behaviors.

Third, this study focused on two moderators—leadership style and network ties—while many other factors, such as team climate, organizational culture, or individual differences (e.g., technological self-efficacy, resilience), may also shape the digital fatigue–engagement link. Exploring such additional contingencies could yield a more comprehensive understanding of how various resources interact to mitigate digital overload.

Lastly, the conceptualization of digital fatigue, though multifaceted, could be further refined. Future research might investigate how different facets of digital fatigue interact with each other or how interventions targeting one dimension (e.g., reducing visual strain) influence others (e.g., emotional or social fatigue). By continuing to dissect and examine the mechanisms of digital fatigue, scholars and practitioners can develop even more nuanced strategies to maintain optimal engagement levels in an increasingly digitalized world.

## Data Availability

The raw data supporting the conclusions of this article will be made available by the authors, without undue reservation.
