# Supplementary material for: Protecting work engagement from digital fatigue: the contingent roles of leadership style and network ties
Source: Front Psychol. 2025 Nov 14;16:1645057. doi: 10.3389/fpsyg.2025.1645057 (PMC12662092; doi:10.3389/fpsyg.2025.1645057)
Supplement: Supplementary file 1 [file Table_1.docx]

**Appendix A:** *Study variables and measurement items*

| Variables | *Items* | | *Factor loading* |
| --- | --- | --- | --- |
| **Digital fatigue**  (Cronbach’s α=0.919;  CR=0.933  AVE=0.5803） | *VF1* | Prolonged periods of working with digital tools make my eyes feel strained or irritated. | 0.800*** |
|  | *VF2* | I often feel eye discomfort or pain due to the amount of time I spend looking at screens. | 0.757*** |
|  | *EF1* | Extended engagement with digital technologies leaves me feeling emotionally drained. | 0.806*** |
|  | *EF2* | Dealing with a constant flow of digital work demands makes me feel emotionally “worn out.” | 0.737*** |
|  | *EF3* | By the end of the workday, my emotions feel exhausted from managing digital interactions. | 0.767*** |
|  | *SF1* | I prefer to be alone after prolonged digital communication sessions. | 0.759*** |
|  | *SF2* | High levels of digital communication make me feel a need to withdraw from social contact. | 0.744*** |
|  | *MF1* | After managing numerous digital tasks, I find it hard to stay motivated for subsequent work. | 0.744*** |
|  | *MF2* | Long periods spent in digital work environments diminish my willingness to take on new challenges. | 0.767*** |
|  | *MF3* | I often have little energy or inclination to engage in work-related activities after prolonged digital exposure. | 0.733*** |
| ***Work engagement***  (Cronbach’s α=0.938;  CR=0.948  AVE=0.6704） | *WV1* | At my work, I feel bursting with energy | 0.817*** |
|  | *WV2* | At my job, I feel strong and vigorous | 0.821*** |
|  | *WV3* | When I get up in the morning, I feel like going to work | 0.792*** |
|  | *WD1* | I am enthusiastic about my job | 0.845*** |
|  | *WD2* | My job inspires me | 0.837*** |
|  | *WD3* | I am proud of the work that I do | 0.786*** |
|  | *WA1* | I feel happy when I am working intensely | 0.785*** |
|  | *WA2* | I am fully immersed in my work | 0.830*** |
|  | *WA3* | I become completely absorbed in my work | 0.853*** |
| ***Transformational Leadership***  (Cronbach’s α=0.938;  CR=0.949  AVE=0.6994） | *TFL1* | He/She encourages employees to think about how to accomplish tasks from a new perspective | 0.84*** |
|  | *TFL2* | He/She encourages employees to consider different viewpoints when analyzing problems | 0.829*** |
|  | *TFL3* | He/She encourages employees to use different approaches to solve problems | 0.829*** |
|  | *TFL4* | He/She speaks optimistically about the future | 0.833*** |
|  | *TFL5* | He/She communicates a clear vision and motivates employees to work hard | 0.799*** |
|  | *TFL6* | His/Her behavior earns employees’ respect and admiration | 0.856*** |
|  | *TFL7* | He/She recognizes that each employee has different needs, abilities, and aspirations | 0.847*** |
|  | *TFL8* | He/She helps employees leverage their strengths | 0.856*** |
| ***Transactional Leadership***  (Cronbach’s α=0.948;  CR=0.960  AVE=0.8282） | *TAL1* | He/She makes it clear to employees what rewards they will receive for achieving goals | 0.911*** |
|  | *TAL2* | He/She provides employees with rewards and assistance to encourage harder work | 0.900*** |
|  | *TAL3* | He/She pays close attention to employees’ noncompliance and exceptional errors | 0.918*** |
|  | *TAL4* | He/She devotes considerable effort to handling deviations, complaints, and mistakes | 0.896*** |
|  | *TAL5* | He/She closely monitors employees’ errors and thoroughly understands problems | 0.925*** |
| **Internal ties**  (Cronbach’s α=0.845;  CR=0.890  AVE=0.6189） | *IN1* | Participants listed up to five close colleagues within their organization. If fewer than five were available, additional colleagues with frequent interaction were listed. Closeness was rated on a five-point Likert scale. | 0.766*** |
|  | *IN2* |  | 0.791*** |
|  | *IN3* |  | 0.806*** |
|  | *IN4* |  | 0.778*** |
|  | *IN5* |  | 0.792*** |
| **External ties**  (Cronbach’s α=0.842;  CR=0.888  AVE=0.6138） | *ER1* | Participants listed up to five external friends (excluding family members) with whom they regularly discussed work and life matters. Closeness was rated on a five-point Likert scale. | 0.752*** |
|  | *ER2* |  | 0.799*** |
|  | *ER3* |  | 0.801*** |
|  | *ER4* |  | 0.796*** |
|  | *ER5* |  | 0.768*** |

*Note*:1. VF= Visual Fatigue, EF= Emotional Fatigue, SF= Social Fatigue, MF= Motivational Fatigue. WV= Vogor ,WD=Dedication, WA=Absorption. 2. *** indicates significance at the 0.00 level.

**Appendix B:** *Results of the EFA*

| ***Variable/ Items*** | | ***Factors*** | | | | | |
| --- | --- | --- | --- | --- | --- | --- | --- |
|  |  | ***1*** | ***2*** | ***3*** | ***4*** | ***5*** | ***6*** |
| ***Digital fatigue*** | *VF1* | -.142 | **0.783** | -.046 | -.116 | .013 | -.041 |
|  | *VF2* | -.084 | **0.756** | .007 | -.003 | -.035 | .043 |
|  | *EF1* | -.035 | **0.811** | -.044 | .008 | .035 | -.008 |
|  | *EF2* | -.124 | **0.727** | -.044 | .041 | .041 | .005 |
|  | *EF3* | -.134 | **0.736** | -.204 | -.060 | -.012 | .018 |
|  | *SF1* | -.160 | **0.727** | -.153 | -.100 | -.038 | .049 |
|  | *SF2* | -.088 | **0.728** | -.095 | -.105 | .082 | .059 |
|  | *MF1* | -.128 | **0.733** | -.046 | -.027 | .010 | -.028 |
|  | *MF2* | -.135 | **0.766** | .056 | .034 | -.033 | -.014 |
|  | *MF3* | -.103 | **0.730** | -.056 | .091 | -.082 | .004 |
| ***Work engagement*** | *WV1* | **0.689** | -.170 | .328 | -.108 | -.108 | .204 |
|  | *WV2* | **0.767** | -.191 | .149 | -.096 | -.044 | .193 |
|  | *WV3* | **0.711** | -.228 | .210 | -.180 | -.022 | .075 |
|  | *WD1* | **0.740** | -.219 | .254 | -.114 | .009 | .205 |
|  | *WD2* | **0.772** | -.083 | .263 | -.103 | -.062 | .169 |
|  | *WD3* | **0.680** | -.152 | .284 | -.057 | .001 | .209 |
|  | *WA1* | **0.672** | -.183 | .343 | -.015 | .020 | .147 |
|  | *WA2* | **0.792** | -.145 | .208 | -.083 | .048 | .103 |
|  | *WA3* | **0.781** | -.142 | .303 | -.076 | .035 | .114 |
| ***Transformational leadership*** | *TFL1* | .303 | -.036 | **0.750** | -.197 | -.110 | .084 |
|  | *TFL2* | .296 | -.080 | **0.751** | -.112 | -.010 | .128 |
|  | *TFL3* | .303 | -.081 | **0.753** | -.113 | -.002 | .099 |
|  | *TFL4* | .209 | -.153 | **0.768** | -.181 | -.019 | .114 |
|  | *TFL5* | .190 | -.007 | **0.750** | -.125 | -.015 | .222 |
|  | *TFL6* | .261 | -.145 | **0.772** | -.171 | -.033 | .138 |
|  | *TFL7* | .281 | -.060 | **0.749** | -.134 | -.020 | .236 |
|  | *TFL8* | .299 | -.112 | **0.764** | -.158 | -.034 | .148 |
| ***Transactional leadership*** | *TAL1* | -.135 | -.042 | -.194 | **0.857** | -.059 | -.169 |
|  | *TAL2* | -.108 | -.031 | -.164 | **0.868** | -.053 | -.117 |
|  | *TAL3* | -.107 | -.037 | -.197 | **0.877** | -.014 | -.148 |
|  | *TAL4* | -.138 | -.067 | -.152 | **0.865** | .032 | -.088 |
|  | *TAL5* | -.114 | -.049 | -.197 | **0.889** | .003 | -.121 |
| ***Internal ties*** | *IN1* | .410 | -.027 | .257 | -.195 | -.086 | **0.548** |
|  | *IN2* | .289 | .058 | .319 | -.121 | .028 | **0.640** |
|  | *IN3* | .278 | .008 | .147 | -.172 | -.046 | **0.725** |
|  | *IN4* | .200 | .033 | .212 | -.046 | -.027 | **0.758** |
|  | *IN5* | .176 | .069 | .186 | -.279 | .030 | **0.726** |
| ***External ties*** | *ER1* | -.005 | .022 | -.074 | -.007 | **0.745** | .045 |
|  | *ER2* | -.032 | -.055 | .048 | .007 | **0.802** | -.097 |
|  | *ER3* | -.064 | -.062 | -.032 | -.009 | **0.799** | .070 |
|  | *ER4* | .011 | .011 | .005 | -.009 | **0.796** | -.016 |
|  | *ER5* | .018 | .062 | -.082 | -.048 | **0.763** | -.063 |

Note: The extraction method was Principal Component Analysis, with Varimax rotation using Kaiser normalization. The rotation converged after six iterations.
